# Detection of Atypical Salmonella Infantis Phenotypes in Broiler Environmental Samples

**DOI:** 10.1128/spectrum.00106-23

**Published:** 2023-05-11

**Authors:** Claudia Hess, Victoria Drauch, Joachim Spergser, Christian Kornschober, Michael Hess

**Affiliations:** a Clinic for Poultry and Fish Medicine, University of Veterinary Medicine Vienna, Vienna, Austria; b Institute for Microbiology, University of Veterinary Medicine Vienna, Vienna, Austria; c National Reference Centre for Salmonella, Austrian Agency for Health and Food Safety, Graz, Austria; Institut National de Santé Publique du Québec

**Keywords:** *Salmonella*, variants, motility, chicken, characteristics, phenotype, hydrogen sulfide, variable phenotypes

## Abstract

In numerous countries, strict and targeted measures concerning Salmonella monitoring and control are implemented and high quality of surveillance is ensured by obligatory investigation of samples from the primary production level of animals according to EN/ISO standards. Here, 2 phenotypic characteristics of Salmonella exhibited on compulsory media are crucial, namely, motility demonstrated on modified semisolid Rappaport Vassiliadis agar (MSRV), and production of hydrogen sulfide (H_2_S) on xylose lysine deoxycholate agar (XLD). In the present study, we describe the detection of Salmonella Infantis variants found in broiler environmental samples with major alterations in their growth characteristics on MSRV, XLD, and brilliant green-phenol red-agar (BPLS). The variants proved to be non-motile on MSRV and displayed non-confirming colony appearances on the previously mentioned selective agars. The growth spectrum comprised pinhead sized yellow colonies with small black centers, but also pinpoint sized colorless colonies, both colony types of regular shape. Our work contributes to highlight the finding of *S*. Infantis variants which possess more than one phenotypic deviation from the “typical” growth characteristics and by this limit the detection power of the actual obligatory used media.

**IMPORTANCE** Salmonellosis caused by non-typhoidal Salmonella serovars is the second most frequently reported zoonotic disease in humans in the EU. The transmission of these agents is mainly via contaminated food of animal origin. In this context, poultry products are the main source of infection. Therefore, continuous and standardized surveillance of the prevalence of such Salmonella serovars at the primary production level is essential. Our findings show the phenotypic heterogeneity of the serovar Infantis and provide growth characteristics of atypical variants. Such variants pass unnoticed official screening methods, resulting in incorrect identification and being underrepresented in epidemiological surveillance programs.

## OBSERVATION

In recent years, Salmonella Infantis became the most common serovar in broiler chickens in the European Union. This goes along with being among the top 4 common serovars in humans (1). Of certain concern are isolates harboring a large conjugative megaplasmid named pESI (plasmid of Emerging *S*. Infantis) which confers antimicrobial resistance and increases the fitness of its bacterial host (2–4). In breeding flocks of Gallus gallus, *S*. Infantis belongs to the target serovars with eradication of positive flocks. This is different to the situation in broiler flocks where carcasses from positive flocks enter the food chain as fresh meat (5, 6). However, logistic measures including slaughtering of such positive flocks at the end of the day are set by the abattoirs. Additionally, further preparation of their fresh meat is not allowed (7). In the EU, strict and targeted measures concerning Salmonella monitoring and control are implemented by legislation (8, 9). To ensure high quality of surveillance, samples from the primary production level of animals are obliged to be investigated according to EN/ISO standards (10). Here, 2 phenotypic characteristics of Salmonella exhibited on compulsory media are crucial, namely, motility demonstrated on modified semisolid Rappaport Vassiliadis agar (MSRV) and production of hydrogen sulfide (H_2_S) on xylose lysine deoxycholate agar (XLD).

**FIG 1:**
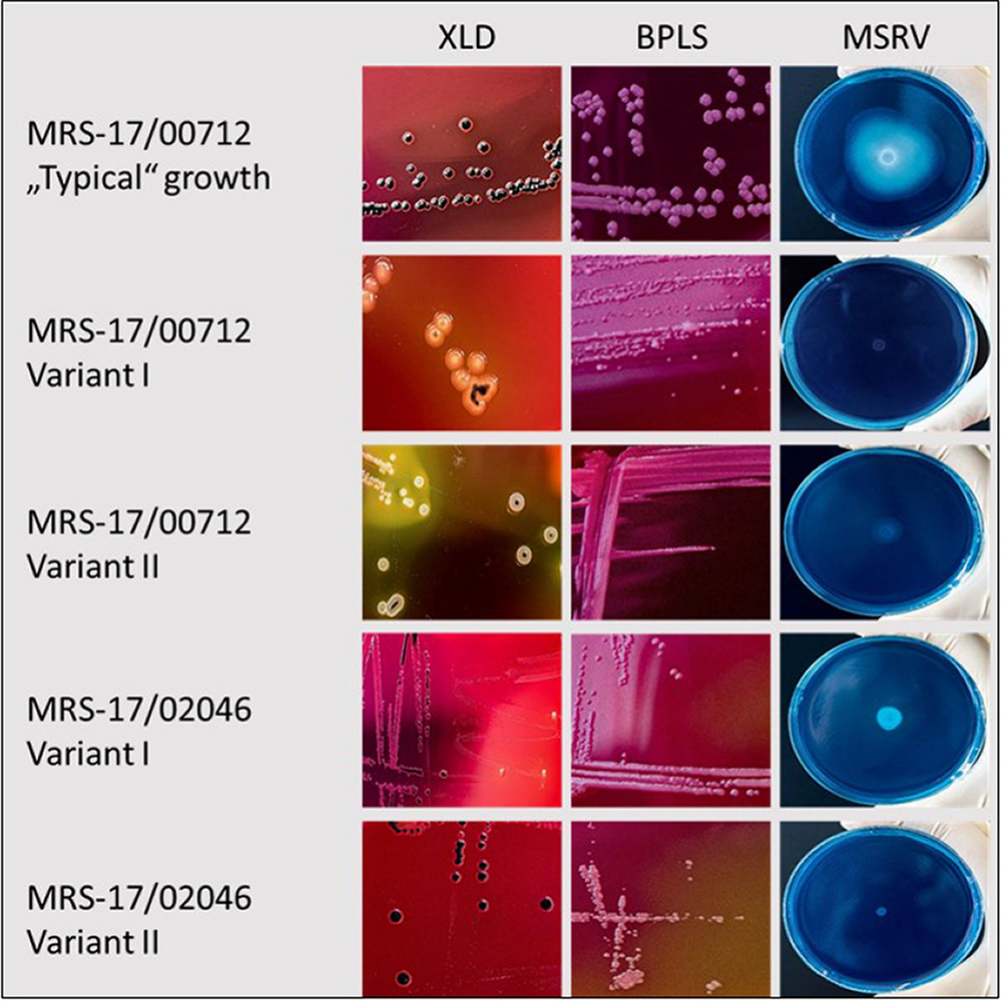
Growth characteristics of MRS-17/00712 and MRS-17/02046 on XLD (24 h), BPLS (24 h), and MSRV (48 h) agar.

Recently, we performed an infection study in chickens using 2 phenotypically different *S*. Infantis isolates (license number GZ.: 68.205/0157-V/3b/2019) (4). Both isolates were obtained as stocks from the National Reference Centre for Salmonella (Graz, Austria). Isolate MRS-16/01939 grew in typical raised black colonies on XLD agar (Merck, Vienna, Austria) whereas isolate MRS-17/00712 presented whitish-yellow colonies with black centers. For the experimental setting, 3 groups of ROSS 308 broilers each comprising 25 birds were housed separately in isolators (Montair HM2500, Montair Environmental Solutions B.V.). Birds from groups 1 and 2 were orally infected with 10^8^ CFU/mL of isolates MRS-16/01929 and MRS-17/00712, respectively. The third group served as negative control, and birds received orally phosphate-buffered saline (PBS, GIBCO). Besides re-isolation of isolates by direct plating on XLD, the enrichment procedure according EN/ISO 6579-1:2017 (10) was applied. During this procedure we recognized that re-isolates from MRS-17/00712 infected birds did not show motility on MSRV. However, they could be cultivated on XLD and BPLS (Bertoni, Vienna, Austria) after transferring material from the inoculum placed on MSRV. This observation prompted us to screen the original stock from isolate MRS-17/00712 together with 20 additional *S*. Infantis stocks derived from different Austrian geographical areas and different broiler flocks for aberrant phenotypes of *S*. Infantis. All isolates originated from boot sock samples taken before slaughter of broilers according to the National Austrian Salmonella Control Program (11). These isolates were stored at −80°C. After thawing, 100 μL were plated on XLD and BPLS agar (aerobic, 37°C) to investigate the colony morphology. Besides colonies of “typical” growth stock MRS-17/00712 presented 2 additional different variants. Variant I, MRS-17/00712-I, presented pinhead sized convex yellow colonies with black centers on XLD and no color change of the agar. Partially confluent pinhead sized reddish-pink colonies with irregular shape occurred on BPLS. Variant II, MRS-17/00712-II, showed pinhead sized flat yellow colonies with small black centers, and viscid appearance on XLD with a color change of the agar from red to yellow. Colonies on BPLS were identical to those of variant I. Of the 20 additional *S*. Infantis stocks, MRS-17/02046 comprised 2 variants. Variant I, MRS-17/02046-I, exhibited convex pinhead to pinpoint sized colorless or black colonies on XLD. On BPLS pinpoint sized reddish colonies of irregular shape, partially with confluent growth were observed. Variant II, MRS-17/02046-II, presented pinhead sized convex black colonies of regular shape on XLD. On BPLS the colony characteristics were identical to variant I. For motility testing, 1 single colony was picked from each type, suspended in 10 mL Luria-Bertani broth (Invitrogen, ThermoFisher Scientific, Vienna, Austria), and incubated at 37°C for 24 h (agitation 120 rpm). One drop of the suspension was pipetted on MSRV, incubated at 41.5°C, and evaluated after 24 h and 48 h. None of the variants showed motility since no swarming zone was observed. The growth characteristics of the cultures are presented in Fig. 1. All variants proved positive for Salmonella group C by slide agglutination test (Sifin Diagnostics GmbH, Berlin, Germany). Determination of serotype was performed according the White-Kauffmann-Le Minor scheme (12). All isolates were identified as *S*. Infantis (antigen formula 6,7: r: 1,5), except MRS-17/00712-II which was attributed as rough form of *S*. I (Salmonella enterica subsp. *enterica*) for which determination of antigen formula was not possible.

The occurrence of atypical Salmonella species has been previously described. These variants were either found to be non-motile or exhibited a lack of H_2_S production (13–20). Bacterial adaptation processes to environmental stress are known to contribute to the evolvement of phenotypic variants. Starvation as well as osmotic or oxidative stress are important inducers for such events (21–23). For *S*. Infantis, it can be hypothesized that intensified cleaning and disinfection procedures which were implemented to eradicate the serovar in the primary production level might have contributed to the emergence of such phenotypic variants.

In conclusion, we report the detection of atypical *S*. Infantis variants from broiler environmental samples which is of serious concern as their detection will be missed applying standard procedures. We describe that several atypical phenotypic growth features can be expressed in 1 variant providing comprehensive data in regard to the aberrations.

### Ethical statement.

The animal trial was approved by the institutional ethics committee and the national authority according to section 8ff of the Law for Animal Experiments, Tierversuchsgesetz (license number GZ.: 68.205/0157-V/3b/2019).
